# IoT-Based Bee Swarm Activity Acoustic Classification Using Deep Neural Networks

**DOI:** 10.3390/s21030676

**Published:** 2021-01-20

**Authors:** Andrej Zgank

**Affiliations:** Faculty of Electrical Engineering and Computer Science, University of Maribor, 2000 Maribor, Slovenia; andrej.zgank@um.si; Tel.: +386-2-220-7206

**Keywords:** bee acoustic analysis, deep neural networks, activity monitoring, acoustic classification, lossy audio compression

## Abstract

Animal activity acoustic monitoring is becoming one of the necessary tools in agriculture, including beekeeping. It can assist in the control of beehives in remote locations. It is possible to classify bee swarm activity from audio signals using such approaches. A deep neural networks IoT-based acoustic swarm classification is proposed in this paper. Audio recordings were obtained from the Open Source Beehive project. Mel-frequency cepstral coefficients features were extracted from the audio signal. The lossless WAV and lossy MP3 audio formats were compared for IoT-based solutions. An analysis was made of the impact of the deep neural network parameters on the classification results. The best overall classification accuracy with uncompressed audio was 94.09%, but MP3 compression degraded the DNN accuracy by over 10%. The evaluation of the proposed deep neural networks IoT-based bee activity acoustic classification showed improved results if compared to the previous hidden Markov models system.

## 1. Introduction

Technology development in the last century has transformed all segments of human society. Artificial intelligence has played an important role in this transformation, as it is crucial for providing the most advanced solutions [1]. The large data amounts, graphic processors, and deep learning algorithms [2] are primary reasons for the rapid development of artificial intelligence. In parallel, due to the embedded systems’ miniaturization and the comprehensive implementation of various networks, there has been a rapid development of the Internet of Things (IoT) [3].

Agriculture is a conservative economic sector, but despite this, we can observe the fast introduction of various Internet of Things solutions in everyday practice [4]. One of the more common tasks in agriculture is the automatic monitoring of animal activity [5], which improves the health and welfare of animals. The working conditions on the farm are also improved [6]. This is even more important when monitored animals are located away from the farm. Beekeeping can be counted in this category [7]. Beehives are often placed in isolated rural locations or even in an urban environment, where rooftop urban beekeeping has gained in popularity. All these locations are remote and difficult for humans to monitor. Despite the location, the bee swarming activity is still a crucial one for the beekeeper. Thus, a bee activity monitoring system can alert the beekeeper about swarming. Various indicators can be used for bees’ activity monitoring, and one of them is sound.

Bees produce different sounds, depending on their activity. The types differ in energy and frequency distribution [8]. The design of an IoT system for acoustic swarm monitoring was presented in [9]. The presented solution could carry out automatic classification of bee swarm activity based on captured audio. Two feature extraction procedures were compared: Mel-frequency cepstral coefficients (MFCC) [10] and linear predictive coefficients (LPC). The classification process was constructed around Gaussian mixture models (GMM) and hidden Markov models (HMM). The latter ones proved to be a better solution for the defined experiment. Many established approaches from automatic speech recognition (ASR) were used in designing the IoT system architecture. In the case of ASR, deep neural networks (DNN) have proven to be an effective solution for pattern recognition tasks [11]. Therefore, introducing DNN acoustic models to the IoT-based automatic bee activities’ acoustic classification is proposed.

The main contribution of this article is to apply the deep neural networks for bee swarm activity acoustic classification. The usage of DNN acoustic modeling approaches from the field of automatic speech recognition is proposed. This represents an efficient solution, which also adds to rapid development. These criteria are essential when considering practical applications. Another important contribution of this article is the analysis of lossy MP3 codec audio compression for bee sounds. MP3 codec is a standard solution applied in the ICT domain [12]. In the IoT systems network design, a distributed topology often appears, where the sound is only captured on the location. For processing, the audio must be transferred over the network to a server. In the case of continuous 24-h beehive monitoring, the amount of audio data transmitted can be significant, so the use of lossy compression is an advantage. Thus, the main article objective is to develop an efficient deep learning acoustic activity modeling technique for uncompressed or lossy compressed bee sounds. 

The paper is organized as follows. A literature review is given in Section 2. Section 3 comprises a description of audio data and the methods used in the paper. Details about the experimental setup are also given in this section. The evaluation of acoustic monitoring with the deep neural networks is reported in Section 4. The discussion is given in Section 5. The paper concludes with Section 6.

## 2. Related Work

Beekeeping is one of the oldest agricultural activities, but the first technological approaches were soon used to monitor bees’ condition in the hive [13]. The earliest experiments monitored various physical parameters of the beehive: temperature [14], mass [15], or humidity [15]. Usage of pattern recognition approaches emerged with the development of artificial intelligence [16]. Acoustic monitoring systems based on bee sound appeared first [16], as such a design is less computationally demanding. With further development of technology, video surveillance systems were also introduced for bees [17,18]. Namely, they need more computing power to operate. The IoT systems development brought the benefits of miniaturization, connectivity, and increased capacity. All these features applied improved the results of bee activity acoustic analysis.

Two main research areas formed in the field of acoustic monitoring. The first one was focused on audio signal feature extraction, and the second one on classifiers and system development. The same algorithms as in automatic speech recognition were often encountered in feature extraction. Linear predictive coding feature extraction [19] was one of the first such approaches used in bee acoustic analysis. Several authors used mel-frequency cepstral coefficients [20,21], which can be considered among the standard procedures for feature extraction. One method used for bee sounds feature extraction was Hilbert-Huang transformation [22,23]. In [23], a more complex approach based on wavelet transformation, was also applied for feature extraction and provided valuable results. 

One of the first approaches for bee sounds’ classification by neural networks was proposed in 2013 by Howard et al. [24]. The authors used a self-organizing map (SOM) ANN. A broader comparison of classification approaches for bee sounds and other acoustic events was made in 2018 by Kulyukin et al. [25]. They compared the use of convolutional neural networks with machine learning algorithms such as k-NN, support vector machine, random forest. Convolutional neural networks with MFCC features were used by the authors in [22]. A more detailed review of bee activity acoustic analysis literature can be found in [26].

A video signal can also be used for bee activity monitoring. Ngo et al. [18] presented such a system, where the video was captured at the beehive entrance. Video monitoring systems were applied frequently for human activity recognition in the IoT smart home environment [27,28].

Animal and human acoustic analysis is related to acoustic analysis in the industrial environment. Similar artificial intelligence solutions were deployed successfully to motors’ acoustic fault detection [29,30]. The authors in [29] proposed a system based on Msaf-ratio-24-Multiexpanded-Filter-8 feature extraction and k-means clustering. The approach achieved recognition results between 95% and 96%.

## 3. Materials and Methods

**Figure 1:**
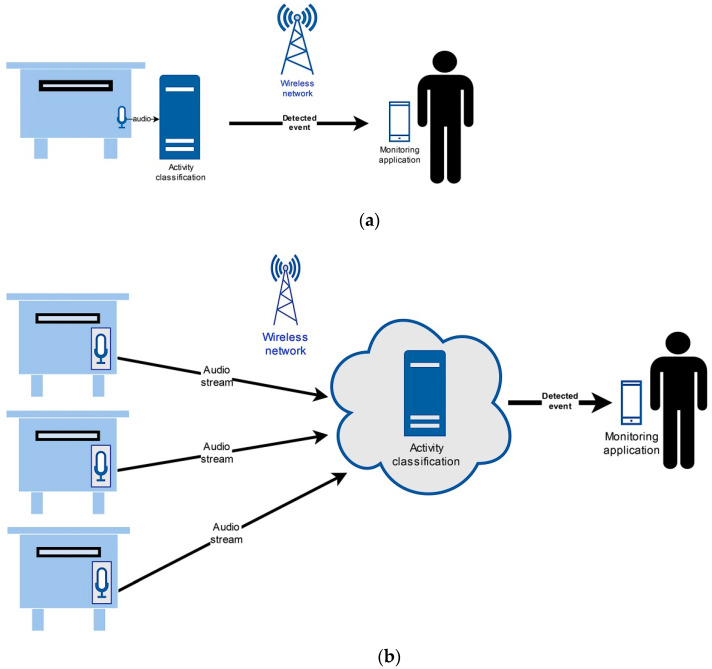
IoT architecture design with a server at the beehive location (**a**) or at a central location (**b**).

If the bee swarm activity acoustic monitoring is designed as an Internet of Things service, then it can be used in various smart environments [9]. One of the fundamental IoT architecture design challenges is where to place the acoustic activity recognition entity. Such a server needs adequate computing power to operate. There are two possible approaches available. In the first one, the server is present directly at the beehive. In the second one, only the audio signal is captured at the beehive location. It is then sent over the network to the server, present at the smart environment’s central location. Such a server can be implemented in the cloud. Both approaches are presented in Figure 1.

A server at the location (Figure 1a) is a more suitable solution for smaller arrangements. As a result, the server may be less powerful. The essential advantage is that audio capture, feature processing, and deep neural network recognition occur in one location. Only the bee activity data are transmitted over the network as information for the beekeeping alarm service in a smart environment [9]. The transferred data amount is so minimal that even the short message service (SMS) in the GSM mobile network can be used if no other technology covers the location. Such architecture has more difficulties with scaling when the beehives number increases. If the computation load limit is reached, a new server must be installed. This increases the operation cost significantly, while also increasing the system’s maintenance and management complexity.

**Figure 2:**
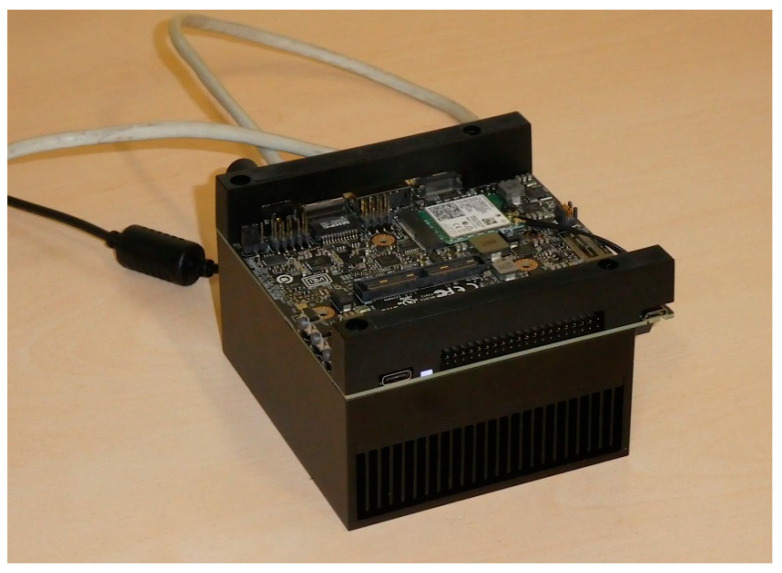
An embedded single board computer for the beehive location.

The IoT architecture with a central server (see Figure 1b) has some advantages over the previous solution. Only the audio signal is captured at the location of the beehive. The captured samples are then transferred over the network to a central location. There, the feature extraction and deep neural network recognition of activities from the audio signal occur. The required hardware at the beehive location can be less complex and with smaller physical dimensions. Therefore, it would be easier to install it in the beehive. An example of an embedded single board computer, which could be used for this task, is shown on Figure 2.

Such embedded single board computers have lower electric power demands. As a result, it is easier to implement an electric power supply with renewable sources in remote locations. This adds to the operation sustainability. Integrating new beehives into the acoustic monitoring system is more manageable. Modules need to be added for capturing and transmitting the audio signal. Processing and DNN recognition are performed at a central location. With the help of virtualization or the infrastructure as a service (IaaS) approach, it is relatively easy to scale the computing capacity. The disadvantage is that an adequately responsive network is required to enable real-time transmission of the audio signal. It is difficult to provide such condition in some remote locations. In the urban beekeeping case this should not be a problem, due to the smart city environment. Since it is necessary to transfer a considerable amount of audio data continuously, the vital question is how effectively this can be done. One solution is to apply lossy MP3 audio compression, used widely for music and speech processing [12]. In case of the beehive location scenario, the needed network bandwidth is only a few kilobits per second, as only the event information must be transmitted. For central location, the bandwidth depends on the audio encoding. If the uncompressed audio is deployed, the default bandwidth is 256 kbps and for the compressed audio it is between 16 kbps and 64 kbps.

The second architecture has an additional disadvantage, as adequate care must be taken to provide sufficient computing capacity. If the required computing power for processing and recognition is distributed, delays can occur. If these are short-lived, they will cause a delay in determining the activity. However, if the lack of computing capacity is more significant, the delays will rise to a level which results in irreversible loss of captured audio data. Consequently, the entire service performance will be impaired. In extreme cases, even the bee swarm activity could stay unnoticed.

### 3.1. Audio Data

The artificial intelligence animal acoustic analysis requires the use of sound recordings collection. Various authors [9,26] have already pointed out the drawback for research of the small amount of bee sound recordings freely available. Particularly problematic is the amount and length of recordings covering the swarm activity. A relevant literature review has shown [26] that there has been no significant improvement recently. The freely available research data amount has not increased. This can present one of the main research field challenges. It is known [1,31] from artificial intelligence and the Internet of Things that open data can stimulate the research area’s development.

Open bee sounds data, generated by the Open Source Beehives (OSBH) project, were used for deep neural networks bee swarm acoustic monitoring system development. The project [32] started with amateurs’ help as an open science initiative. Their goal was to improve the beekeeping with inclusion of advanced IoT technologies. Beekeepers around the world recorded bee sounds in their hives. The beehive type, recording equipment placement, and microphone orientation varied between beehives and locations. Also, different recording equipment was used, depending on the availability. All these characteristics had an influence on the audio signal and its quality. Most recordings were captured with a 44.1 kHz sampling rate, 16 bit, stereo, or mono. There was also no uniform standard format for collecting audio files. Thus, some files were stored in a lossless format (e.g., Microsoft WAV), and others in a lossy format (e.g., MP3). It is known from the automatic speech recognition [33,34,35] that lossy audio compression can worsen the speech recognition results in the 1% to 3% range. The complete unprocessed OSBH audio database resulted in over 48 GB of data. The audio recordings collected with general public involvement eased and shortened the data collection step. However, the disadvantage of such an approach is reflected in its diverse and challenging audio conditions.

The OSBH database source audio recordings were down-sampled to 16 kHz, 16 bit, mono in a lossless WAV format. Bee sound recordings prepared in the OSBH project are available for research on the Zenodo open data sharing platform [36]. The recordings cover the main bee activity categories: normal and swarm. Since there are significantly fewer recordings with swarm activity, the recordings amount was balanced in both categories. Namely, a balanced training corpus size was needed to learn the deep neural networks parameters. The selected recordings were cut into homogeneous parts, where each one was 3 s long. During the initial development, 2-s and 4-s long recordings were also deployed for acoustic modeling. However, the obtained bee acoustic monitoring results were lower. Therefore, further experiments were skipped with them. Balancing the normal activity recordings’ number was then performed by selecting candidates randomly from the initial set. Thus, the final amount of training material comprised 90 min of recordings. Another 32 min of recordings were prepared similarly for testing. It is also necessary to prepare labels for successful deep neural networks learning. These labels are equivalent to transcriptions in the case of automatic speech recognition. Labels were prepared using the OSBH database information about the beehive condition in each recording. The prepared data were then used for learning and testing deep neural networks for bee activity acoustic monitoring.

### 3.2. Neural Networks for Bee Activity Acoustic Monitoring

Deep neural networks are today a widely used architecture for artificial intelligence systems. The idea behind neural networks is to mimic a human neuron’s model and its function of connecting with neighboring neurons. Their design originates from the 1950s [37,38]. The general architecture consists of layers, where the first one represents the input layer and the last one the output layer. Deep neural network architecture has two or more hidden layers with a weight matrix (*W*). The input vector and the weight matrix are multiplied, and then used with the activation function (*af*), defined as:

(1) $$h_{i}^{n}=af\left(\sum_{j}h_{j}^{\left(n-1\right)}W_{i,j}^{\left(n\right)}+b_{i}^{n}\right)$$

where *h* denotes the output, *i* and *j* the particular unit, *n* and *n*−1 the particular layers, and *b* the bias vector. Different activation functions can be used in the architecture: hyperbolic tangent (*tanh*), rectified linear unit (*ReLU*), scaled exponential linear unit (*SeLU*), p-norm, logistic sigmoid, etc.

Deep neural networks have experienced fast development, especially in the last decade [11]. This is due mainly to the availability of big data and advances in hardware. Deep neural network parameters’ calculation is performed by dividing learning onto multiprocessor or multicore systems. The current approach uses graphics processors (GPUs) as multicore systems to learn deep neural networks. GPUs can have several 1000 available parallel cores, which can be used for learning the DNN parameters.

Deep neural network development occurs mainly in speech technologies [11], image processing, and natural language processing fields. Such approaches and solutions can also be used in other artificial intelligence areas. Some neural network architectures are as follows:

- Feed-forward networks,
- Time-delay neural network (TDNN),
- Recursive neural networks (RNN),
- Long short-term memory (LSTM),
- Convolutional neural networks (CNN).

By increasing the deep neural network architecture’s complexity, a larger training data amount and more powerful hardware are needed for learning the model’s parameters. In scenarios where we want to recognize a smaller set of events/activities, more complex architectures rarely bring better results.

Comparing deep neural networks bee activity acoustic monitoring with the previous GMM and HMM approaches [9] shows that the deep neural networks solution is more similar to the HMM approach. Namely, the DNN output is a complete recognition result for the entire input recording. Therefore, there is no need to perform additional post-processing of results, as with GMM models. This results in a less complex system architecture.

### 3.3. Experimental System

**Figure 3:**
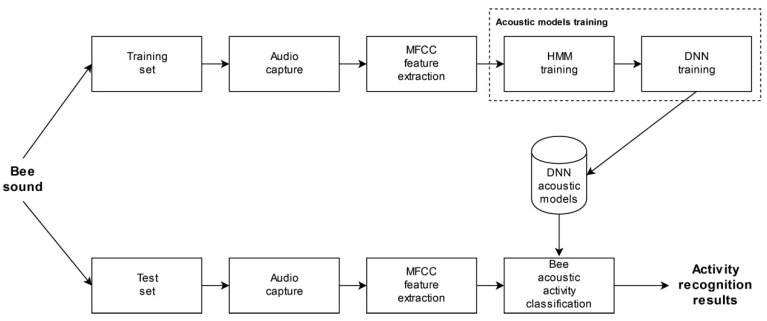
DNN-based bee activity acoustic classification block diagram.

A typical acoustic classification system has the following elements: audio signal capture, feature extraction, acoustic models, and decoding. The block diagram of the proposed DNN-based bee activity acoustic classification system is shown on Figure 3.

More details about them will be given in the following subsections. The experimental system was designed with the Kaldi open-source toolkit [39], used widely in the speech recognition community.

#### 3.3.1. Feature Extraction

The bee acoustic activity database’s audio signal must be prepared before feature extraction. The OSBH audio recordings were first trimmed into homogeneous parts. Then, windowing was carried out with a 25 ms long Hamming window. The window shift was 10 ms. Audio signal pre-emphasis was performed in addition. The feature extraction process was started with the sound signal processed in this way. The results achieved with our previous acoustic monitoring system [9] indicated the use of mel-frequency cepstral coefficients for feature extraction. This feature type is used frequently in the automatic speech recognition, and other audio signal processing areas. The MFCC feature extraction had a filter bank with 26 individual elements. The MFCC feature vector, with 12 elements and energy value, was used in the first acoustic modeling training step. The first and second order derivatives were added in the further acoustic modeling training steps. Thus, the final feature vector had a total of 39 elements. The derivatives contributed to better acoustic modeling of the audio signal over time.

#### 3.3.2. MPEG-1 Audio Layer III Audio Compression

Lossy audio compression was used in order to optimize the amount of bee sound data transmitted over the network to the processing server. The MPEG-1 Audio Layer III format (denoted as MP3) [12] was applied, defined in the ISO/IEC 11172-3:1993 and ISO/IEC 13818-3:1995 standards. The MP3 codec is a widely used format in various areas related to digital audio processing. It is the de facto default standard for using and downloading music over the Internet. Many digital end devices support it, from portable music players and smartwatches to smartphones. It is also widely present in the Internet of Things, for example, in streaming music services, or using virtual assistants in a smart environment. The successor to the MP3 is the advanced audio coded (AAC) codec. It is part of the MPEG-2 Part 7 codec family, and is formalized as an ISO/IEC 13818-7:1997 standard. Using the same bit rate as the MP3 format achieves better sound quality after the lossy compression. The AAC audio compression also allows higher sampling frequencies, up to 96 kHz. However, such high-quality capturing exceeds our experimental setup. It is typically required for high definition audio media content.

The MP3 codec is based on different psychoacoustic effects, which influence humans’ perceptual sensing of sound [40,41]. Any human hearing-inaudible information in the generated sound can be omitted from the signal. The MP3 codec addresses the surplus audible information in the time and frequency domain. It is using sound masking and other digital signal processing approaches based on perceptual psychoacoustic modeling. The encoding procedure can be applied with variable or constant bitrate. A ten times compressed MP3 encoded audio file can be achieved, without significant perceived quality loss.

**Figure 4:**
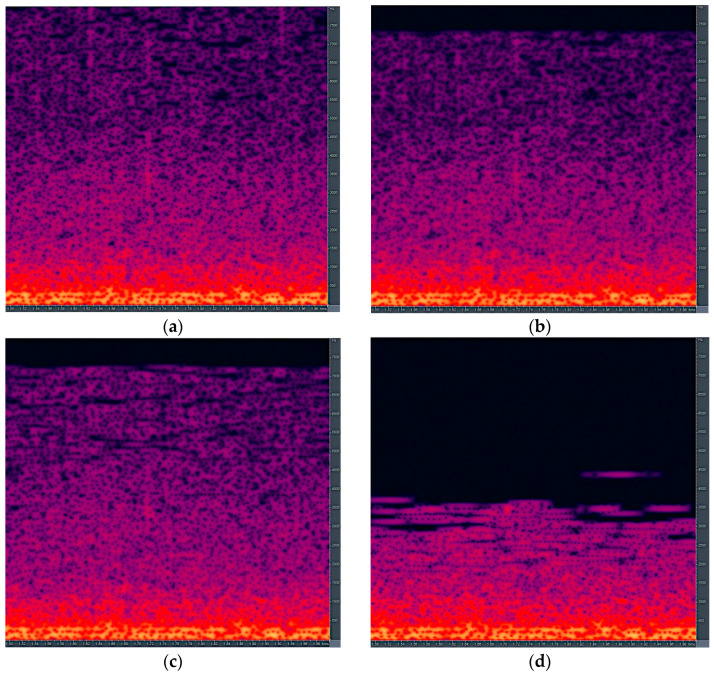
Spectral characteristics of the uncompressed bee audio signal (**a**) and MP3 encoded audio with 64 kbps (**b**), 32 kbps (**c**), and 16 kbps (**d**) bitrates. The X-axis denotes time in seconds and Y-axis denotes frequency in Hz. The amplitude is given by color, where dark represents low amplitude and bright yellow represents high amplitude.

The LAME MP3 encoder with constant bitrate was used for the bee audio signal. Figure 4 shows the uncompressed bee audio signal’s spectral characteristics and three different MP3 encoded bee audio signals. The MP3 bitrates used for bee audio in Figure 4 were 64 kbps, 32 kbps, and 16 kbps, respectively. The average data compression factors were 3.8, 7.7, and 15.3, respectively. A 10-time data compression factor similar to the case of music, is accomplished with 24 kilobits per second bitrate.

The difference in the spectral domain between the uncompressed and the three MP3 encoded bee audio signals shows the artifacts produced by lossy compression. They can be found in the signal after an MP3 encoding procedure. The most notable difference is the cut-off frequency decrease in the high-frequency range. It shifts from approx. 7.8 kHz to 5.5 kHz as the bitrate reduces. Another compression artifact in the spectral domain is the reduction and energy shift in different signal parts. This results from the performed masking approach. The described spectral domain compression artifacts are only the ones which can be seen easily. There are other lossy effects present in the encoded bee audio signal. They are not directly observable, but influence the overall encoded audio quality with the MP3 format.

A thorough analysis of MP3 lossy format was carried out on the results of automatic speech recognition in [33]. In the case of an MP3 encoded spoken signal, the speech recognition accuracy decrease can be in the range of several percent, depending on its characteristics. It is expected that similar results will be observed with bee audio signals, at least for high and medium bitrates.

#### 3.3.3. Training of Acoustic Models

The experimental system was designed using the Kaldi toolkit [39]. It was developed primarily for the automatic speech recognition research. With the modification of support tools, it can also be used to develop an audio classification system. In addition, it can also recognize various sound categories or characteristics. A necessary step is to redevelop the preprocessing tools to prepare data for the learning process. The next necessary step is to design the acoustic models’ training procedure. This depends on the acoustic models’ usage scenario.

After the audio signal was preprocessed and MFCC feature extraction was completed the acoustic models’ training procedure was started. The basic training approach was designed in a way that it was first necessary to train the HMM acoustic models. These served as a predecessor for learning the deep neural networks. The primary goal of HMM acoustic models was to prepare audio signals’ aligned transcriptions efficiently. These were needed during the deep neural networks’ learning process.

Training HMM began with acoustic models combined with an MFCC feature vector that had only 13 core elements. The first and second-order derivatives were omitted. Several iterations of training were repeated. The next step was training the acoustic models, where the MFCC features were included with all 39 elements. In the next step, it was possible to introduce an adaptation to individual recordings or a group of recordings using the Speaker Adaptive Training (SAT) algorithm. This step was excluded in the case of bee acoustic activity classification. Preliminary experiments showed that such adaptation impaired recognition performance with bee sound.

Deep neural networks learning to recognize bee audio activity was started in the next step. A feed-forward neural network with a p-norm activation function was chosen. It is defined as:

(2) $$y={\left\|x\right\|}_{p}={\left(\sum_{i}{\left|x_{i}\right|}^{p}\right)}^{\frac{1}{p}}$$

where input groups are denoted as *x* and *p* denotes the configurable parameter of a non-linear function. The acoustic model’s parameters’ initial values were set based on the training material amount. Two hidden layers were used for the baseline deep neural networks acoustic model. The p-norm based hidden layer input dimension was set to 4000 and output dimension to 400. The p-value of the activation function was set to 2. The initial learning rate was set to 0.02. During the training epochs, it was decreased exponentially to 0.004. The initial number of training epochs was 13. These were default parameters initialized according to the Kaldi automatic speech recognition recipes and modified to the training set size. The basic idea for selecting the parameters was to transfer ASR principles to the bee sound recognition. Several training experiments of deep neural network acoustic models were repeated as part of the analysis of parameters’ influence. Parameter values were optimized in an empirical way, using the Kaldi ASR guidelines and the evaluation result based on a random test subset. These experiments showed that the hidden layer input dimension of 3000 and output dimension of 300 were optimal in combination with the learning rate. The default number of training epochs was changed between 40 and 5 in steps of 5. With 40 epochs, the DNN models were overtrained and the accuracy decreased by approximately 10%. Experiments showed that the optimal number of training epochs was 20. This setup was then used during the evaluation process.

The influence of lossy MP3 coding was modeled as a particular part of the bee activity acoustic monitoring system’s experimental design. The deep neural networks’ acoustic models’ learning process defined above was carried out with MP3 encoded sound.

In the last step of the experimental design, different acoustic models were evaluated with the same bee acoustic activity test set. Different system configurations were compared and analyzed as a result.

## 4. Results

The bee activity acoustic monitoring evaluation was carried out on a 32-min test set containing 643 individual recordings excluded from the training set. The baseline test set had 322 recordings for normal condition and 321 for swarm condition. Each recording was 3 s long. The results are given in the form of accuracy, which is defined as:

(3) $$Acc\left(\%\right)=\frac{H}{N}\cdot 100$$

The correctly recognized bee activity events are denoted as H, and the number of all activity events as N. Accuracy is a typical metric used in automatic speech recognition. However, when such architecture applies to a classification task, the accuracy cannot be the only metric used for the evaluation. Namely, there is a lack of information about individual false positive or negative errors. Therefore, the precision (*P*), recall (*R*), and F1-score metrics were used. The F1-score is defined as:

(4) $$F1=2\cdot \frac{P\cdot R\;}{P+R}=\frac{TP}{TP+\frac{1}{2}\left(FP+FN\right)}$$

where *TP* denotes true positive, *FP* false positive, and *FN* false negative cases in the results. The first evaluation step started with audio classification using the HMM acoustic models. Those served in the development process as a necessary preparation step for learning the deep neural networks. These results served primarily to evaluate the baseline and compare the proposed approach with the previous acoustic monitoring system [9]. The latter was based exclusively on GMM and HMM acoustic models. The results are presented in Table 1.

The new acoustic monitoring system achieved 88.02% accuracy at a 0.936 F1-score with HMM acoustic models. The precision was 0.998 and recall 0.882. Results show that HMM acoustic models’ complexity had only a small influence on accuracy. The best result was significantly better than the one obtained with the previous system [9], where the best result was 82.27%. Both setups’ system complexity could not be compared directly, but it was similar. It can be concluded that the accuracy increase was mainly because of the improved HMM acoustic models’ training procedure provided by the Kaldi toolkit. In comparison with the previous procedure, there were more training steps carried out, but with smaller training iteration sizes. Results were a good baseline for the forced realignment procedure, which was mandatory for training the deep neural network acoustic models. The second evaluation step was focused on deep neural networks’ acoustic models. The evaluation results are given in Table 2.

The deep neural network acoustic models with default two hidden layers achieved 92.85% accuracy and a 0.963 F1-score. The precision was 0.987 and recall 0.940. A higher number of hidden layers increased the accuracy. The best overall experimental result was reached with 4 hidden layers. The accuracy was 94.09%, the F1-score 0.970, and precision and recall were 0.995 and 0.945, respectively. The best DNN accuracy increased by 6.07% compared to the best HMM acoustic models. This presented a significant DNN acoustic models’ performance improvement. The F1-score improved from 0.936 with the HMM acoustic models to 0.970 with the DNN acoustic models. It was mainly the result of increased recall, which indicated a false negative reduction. The probable reason for improvement was that DNN could model the swarm acoustic condition better. This condition had challenging audio characteristics.

The confusion matrix presenting both categories of monitored activities (normal—NOR, swarm—SWM) with the best performing 4 hidden layers DNN acoustic models is given in Table 3.

The best DNN acoustic models hypothesized (NOR_HYP_) 319 normal recordings correctly out of 322, and 286 swarm recordings (SWM_HYP_) out of 321. In the case of normal recordings only three false positives occurred, while on the other hand, the number of false negatives was higher, with 35 occurrences. This indicated additional room for future improvement.

Detailed analysis of misrecognized recordings was carried out. An important finding was that approx. 20% of misrecognized cases had acoustic recordings in which the bee came into direct contact with the microphone housing. This was placed inside the beehive. In most cases, the recording equipment captured bees’ sounds without direct contact with them. Sometimes, however, one or more bees came into direct contact with the microphone housing. Thus, the captured sound had a significant increase in sound energy, which also often ended with degradations. Some were included in the training set. However, according to the analysis, this category probably deviated significantly from the typical acoustic environment captured in deep neural networks acoustic models. Therefore, this test set part contributed a higher errors’ proportion. In future research, it will be necessary to pay special attention to this acoustic events category. The objective will be to improve the bee activities’ acoustic monitoring results further.

The last evaluation step was focused on bee audio processed with MP3 codec. In the first experiment, only the baseline test set of 643 recordings was encoded to MP3 and back. The accuracy degraded drastically. Thus, the complete training procedure was repeated for each MP3 bitrate. The evaluation results are given in Table 4.

Applying MP3 encoding/decoding to the bee audio had a different impact on HMM and DNN acoustic models. The accuracy decrease of HMM acoustic models was in the expected range. The accuracy decreased from 88.02% with uncompressed audio (256 kbps) to 85.23% with 16 kilobits per second. With 32 kbps MP3 bitrate, the HMM accuracy decreased by only 0.46%. The data rate needed for transmission was reduced by factor 8, without a significant decrease of accuracy. On the other hand, the DNN acoustic models performed much worse. The accuracy lowered from 94.09% (uncompressed) to 82.58% (24 kbps), which was the worst overall result. The DNN acoustic models with 24 kbps bitrate were even worse than the HMM acoustic models. Already the small MP3 compression rate, by a factor of 4 (64 kbps), decreased the DNN accuracy by 4.98%. These DNN results showed that the MP3 codec might not be the best solution for some IoT bee acoustic activity classification setups. The probable reason could be that the MP3 psychoacoustic model provided lossy compression results too narrowly oriented on music. The acoustic characteristics of animal sound differed to a greater extent from general music. DNN acoustic models proved to be a suitable solution for uncompressed audio, but they lost one part of their modelling capabilities because of the unmatched audio conditions.

Last part of the evaluation was the computational load analysis for the DNN classification. The classification experiments were carried out on an Intel Xeon CPU at 2.2 GHz. Such a processor type could be used either at the beehive or central location (see Figure 1). Only one core was used for the task. The DNN acoustic models with four hidden layers operated with 0.014 real-time factor. Also, the MP3 encoding computational load was measured. This part was carried out on an ARM v8.2 64-bit CPU single board embedded system. The MP3 encoding procedure real-time factor was 0.011.

## 5. Discussion

The proposed DNN acoustic models improved the bee swarm activity results. The modification of the number of hidden layers was carried out. It has been shown that the bee activity acoustic monitoring accuracy could be improved further in this way. The achieved accuracy was comparable to results of other systems with similar complexity [20,23,25,26]. The hidden layer parameter variation also influenced the complexity of the acoustic models. As a result, the processing time may increase. The entire bee activity acoustic monitoring system was relatively undemanding in terms of the required computational power compared to the state-of-the-art automatic speech recognizers. Because of this difference, the increased bee activity acoustic monitoring complexity did not pose a significant obstacle.

Also, the influence of lossy MP3 encoding on the system’s accuracy was analyzed. The HMM bee activity acoustic monitoring results deteriorated in the range from 1% to 3%. The deterioration with DNN acoustic models was between 4% and 12%. The decrease with DNN was higher than typical for MP3 automatic speech recognition. Nevertheless, the achieved HMM result is still acceptable for a smart environment. One probable explanation for the differences may be that the focus of the MP3 codec is on the human psychoacoustic characteristics present in the speech signal. These, of course, differ from the characteristics found in the bee sounds. Achieving an accuracy level suitable for practical use is vital for the proposed Internet of Things smart environment design. In the beehive, only the audio signal is captured. It is then transmitted over the network to the cloud server for activity recognition. The required bandwidth can be reduced if MP3 lossy compression is used instead of the common loss-less WAV format. This is beneficial, as less powerful wireless technologies can be used. Also, the amount of data transferred is reduced. This can be an essential criterion when using commercial mobile networks.

Another important result is the finding that bees in direct contact with the microphone housing cause the results’ deterioration. This can be avoided by a future appropriate recording system design inside the beehive. For existing, already captured recordings, the future research should focus on modeling approaches that could reduce this effect.

## 6. Conclusions

The proposed system for bee activity acoustic monitoring with DNN acoustic models improved the results compared to the previous solution with HMM. Additional improvement resulted from modifying the number of DNN hidden layers. The best overall recognition accuracy was 94.09%. Lossy MP3 audio codec analysis showed varying performance under degraded conditions. The recognition accuracy decrease was as high as 11.51%, depending on the bitrate and models used.

Future work will focus on other types of deep neural networks and deep learning approaches for bee activity acoustic modeling. Other different DNN architectures, which have proved successful in other pattern recognition tasks, will be included in this future work. Another possible future research direction will be to carry out an impact analysis of other lossy audio codecs known from the ICT domain. They could reach an even more significant reduction in the required transmission bandwidth. Such future work could improve further the everyday usage of bee activity acoustic monitoring as an IoT distributed setup.

## Figures and Tables

**Table 1:** Results of acoustic monitoring of bee audio with HMM acoustic models.

Acoustic Models’ Complexity	Acc. (%)	P	R	F1
Low	86.47	0.998	0.866	0.927
Mid	88.02	0.998	0.882	0.936
High	87.56	0.993	0.881	0.934

**Table 2:** Bee activity acoustic classification results with deep neural network acoustic models.

DNN Models’ Hidden Layers	Acc.	P	R	F1
2	92.85	0.987	0.940	0.963
3	93.16	0.997	0.934	0.965
4	94.09	0.995	0.945	0.970
5	93.16	0.997	0.934	0.965
6	93.47	0.997	0.938	0.966
7	93.16	0.990	0.940	0.965

**Table 3:** Monitored activities’ confusion matrix for DNN acoustic models with 4 hidden layers.

	NOR_REF_	SWM_REF_
NOR_HYP_	319	35
SWM_HYP_	3	286

**Table 4:** HMM and DNN acoustic models bee activity acoustic classification accuracy with MP3 processed audio.

MP3 Bitrate (kbps)	HMM	DNN
uncompressed	88.02	94.09
64	86.63	89.11
32	87.56	90.05
24	87.40	82.58
16	85.23	87.56

## Data Availability

The data presented in this study are openly available in Zenodo at doi:10.5281/zenodo.1321278, reference number 1321278.
